# Impact of two COVID-19 lockdowns on HbA1c levels in patients with type 2 diabetes and associations with patient characteristics: a multicentre, observational cohort study over three years

**DOI:** 10.3389/fpubh.2023.1272769

**Published:** 2024-01-05

**Authors:** Ingmar Schäfer, Daniel Tajdar, Laura Walther, Lasse Bittner, Dagmar Lühmann, Martin Scherer

**Affiliations:** Institute and Outpatients Clinic of General Practice/Primary Care, University Medical Center Hamburg-Eppendorf, Hamburg, Germany

**Keywords:** COVID-19 lockdown, HbA1c, glycemic control, atherosclerotic cardiovascular disease, insulin treatment

## Abstract

**Introduction:**

Glycemic effects of COVID-19 lockdowns on patients with type 2 diabetes (T2D) are controversial. In this long-term observation, we aimed (1) to analyze changes in HbA1c levels during lockdowns in Germany, and (2) to investigate whether diabetes medication, comorbidities, and sociodemographic data influenced these changes.

**Materials and methods:**

This cohort study observed 1,089 patients aged ≥18 years over the years 2019 to 2021. Patients were recruited from 14 physicians specialized on diabetes. As dependent variable, 7,987 HbA1c values were analyzed by multivariable linear regression adjusted for random effects of physicians and patients.

**Results:**

Patients had a median age of 68 (60/76) years and 623 (57.2%) were male. Before the pandemic, median HbA1c level (in %) was 6.9 (6.3/7.7). Average HbA1c level increased during first lockdown (0.21,0.11/0.31,*p* < 0.001), after first lockdown (0.23,0.18/0.28,*p* < 0.001), during second lockdown (0.40,0.33/0.47,*p* < 0.001) and after second lockdown (0.27,0.18/0.36,*p* < 0.001). The increase of HbA1c levels was more pronounced in male patients (0.08,0.01/0.15,*p* = 0.019), if patients did not have German as native language (0.12,0.01/0.23,*p* = 0.041) and if they were widowed (0.19,0.05/0.32,*p* = 0.008). End organ damages (0.12,0.01/0.23,*p* = 0.039), atherosclerotic cardiovascular disease (ASCVD; 0.23,0.10/0.36,*p* = 0.001) and cardiovascular events (0.25,0.10/0.40,p = 0.001) as well as oral medication (0.09,0.03/0.15,*p* = 0.002), intermediate- or long-acting insulins (0.24,0.16/0.32,*p* < 0.001), and fast-acting or mixed insulins (0.30,0.23/0.36,p < 0.001) were also related to a greater increase in HbA1c levels.

**Conclusion:**

Both lockdowns resulted in a significant increase in HbA1c levels. In particular, patients with ASCVD, cardiovascular events, and insulin therapy appear to be at risk for worsening glycemic control in crisis and thus require special medical attention.

**Clinical Trial Registration:**

ClinicalTrials.gov (NCT04821921).

## Introduction

In 2020, the COVID-19 pandemic spread worldwide (1). In order to reduce the incidence of the virus, nationwide lockdowns were implemented in most countries (2, 3). In Germany, a first lockdown was imposed from 22 March to 6 May 2020 and a second lockdown from 13 December 2020 to 30 June 2021 (2, 3). During lockdowns, people were encouraged to stay at home, public life was restricted and planned treatments in hospitals were postponed (2, 4). Also, utilization of outpatient health care declined as many patients were concerned about the high infection rate and mortality risk associated with COVID-19 (2, 4).

Indeed, people with type 2 diabetes, high HbA1c (glycosylated hemoglobin A1c), and comorbidities such as coronary artery disease and hypertension had an increased risk of death after COVID-19 infection (5). In Hamburg, a German metropolitan area, 8.1% of the population (9.0% men and 7.5% women) receive outpatient treatment for type 2 diabetes mellitus (6). The prevalence of diabetes increases with age and the most deprived districts of Hamburg have the highest proportion of patients with type 2 diabetes (6).

The lockdowns may have led to adverse health outcomes, particularly in patients with diabetes mellitus (DM), as physical inactivity and lack of follow-up consultations can result in worsening glycemic control (7) and thus increased HbA1c. For example, the UK Prospective Diabetes Study (UKPDS) found that in the HbA1c range between 5.5 and 10%, the risk of myocardial infarction and diabetes-related deaths increases almost linearly (8). For example, for every 1% increase in updated mean HbA1c, a 14% higher risk of myocardial infarction and a 21% higher risk of diabetes-related death were demonstrated during the study (8).

Several studies investigated the glycemic effects of lockdown on patients with diabetes. However, most studies of the glycemic effects of lockdown were conducted in patients with type 1 diabetes (T1D), although 90 to 95% of patients with diabetes are type 2 (T2D) (7, 8). The studies investigating the association between lockdowns and T2D came to contradictory results. Some studies found an increase in HbA1c levels in T2D patients (9–18), while others found no change or even improvement in glycemic control (1, 19–24).

One explanation for this conflicting evidence could be the relatively short observation period after lockdown. In most studies, Hba1c was measured early at the end of the lockdown period and then compared with HbA1c levels before lockdown (10, 11, 14, 19–22, 24). To the best of our knowledge, there is no published long-term observation of HbA1c after COVID-19 lockdown, yet. This is important, because HbA1c changes slowly over a period of 8 to 12 weeks as its concentration depends on both blood glucose and erythrocyte lifespan, which is approximately 120 days (25). Hence, a longer observation period after lockdown would have been required to properly assess its effect on HbA1c. In addition, few studies considered that HbA1c levels can be influenced by other variables besides lockdown, such as socio-economic status (SES) (26), regional deprivation (27), social support (28), and particularly diabetes medication (29).

Therefore, we conducted this multicenter, observational cohort study over three years to examine the glycemic effects of two lockdowns on T2D. The aim of this study was (1) to analyze if lockdown phases of the COVID-19 pandemic were associated with increasing HbA1c levels, and (2) to examine if patients’ diabetes medication, diabetes-related comorbidities, and socio-demographic data are mediating the change in these levels.

## Materials and methods

### Design, study population and data set

We conducted the longitudinal observational study CoDiaM (“impact of COvid-19 pandemic on DIAbetes Management”), which was based on data extraction from patient records in general practices supplemented by a postal survey. The study was registered in ClinicalTrials.gov (NCT04821921) and approved by the “Local Psychological Ethics Committee at the Center for Psychosocial Medicine of the University Medical Center Hamburg-Eppendorf” on 19 January 2021 (Approval-No. LPEK-0243).

Patients were recruited from three cooperating practices specialized on the treatment of patients with diabetes, in which a total of 14 physicians participated in our study. All patients from the respective physicians were screened for eligibility. We included patients if any diagnosis of diabetes was documented in the records, if they were at least 18 years old, and if they had at least one contact with the attending physician from the participating practice in 2019 and in 2020, respectively.

We excluded patients with gestational diabetes or if they had deceased before recruitment. Other exclusion criteria comprised the change of the attending diabetologist and lack of a valid postal address. Moreover, patients were excluded if they could not read and write or had insufficient German language skills, no capacity to consent or functional limitations precluding participation in the survey.

Eligible patients were contacted by mail and invited to participate in the study. They received an envelope with standardized questionnaire, patient information about the study, and a consent form facilitating data extraction. Time and place in which the questionnaire was filled out were chosen by the patient. Patients were excluded retrospectively if they had type 1 diabetes or pancreoprive diabetes.

Patient recruitment and postal survey were conducted between 22 February and 6 April 2021 for practice 1, between 2 June and 4 August 2021 for practice 2 and between 30 November 2021 and 5 April 2022 for practice 3. The survey included the socio-demographic data age, sex, marital status, native language and general and vocational education. The information about education was used to assign patients to three hierarchical educational levels pursuant to the CASMIN (“Comparative Analysis of Social Mobility in Industrial Nations”) classification, ie, tertiary, secondary and below (30).

For all patients who had given written consent, diagnoses of cardiovascular risk factors such as hypertension, atherosclerotic cardiovascular disease (ASCVD) such as chronic ischemic heart disease, end organ damages such as retinopathy, cardiovascular events such as myocardial infarction, and heart failure were extracted from patient records. We also extracted prescriptions for diabetes medication and results of blood tests commissioned by the practice in the time frame between 1 January 2019 and 31 December 2021 and indicating HbA1c levels.

### Statistical analyses

Descriptive data of the sample were described by fractions and medians with interquartile ranges, respectively. Median HbA1c levels per month and phase of the COVID-19 pandemic were shown for each month with more than 100 observations. As phases we defined the times (1) before first lockdown in Germany (1 January 2019 to 21 March 2020), (2) during first lockdown (22 March 2020 to 6 May 2020), (3) after first lockdown (7 May 2020 to 12 December 2020), (4) during second lockdown (13 December 2020 to 30 June 2021), and (5) after second lockdown (1 July 2021 to 31 December 2021).

Associations between independent variables age, sex, marital status, native language, educational level, diabetes-related comorbidity, prescribed diabetes medication in the 90 days preceding the blood test and the dependent variable HbA1c level were analyzed by longitudinal mixed-effects multivariable linear regression. The analysis was controlled for random effects on physician and patient within physician level, adjusted for the patient’s HbA1c level in the measurement directly preceding the analyzed observation and controlled for number of the respective measurement and phase of COVID-19 pandemic.

We compared a model comprising only observations before the COVID-19 pandemic with a model comprising only observations during the COVID-19 pandemic. Additionally, we conducted unadjusted longitudinal analyses for the independent variables mentioned above. These analyses were also controlled for random effects on practice and patient level, adjusted for the previous HbA1c levels and controlled for number of measurement and phase of pandemic. Additionally, the distribution of continuous variables was analyzed by kernel-density estimation and a correlation matrix between independent variables was calculated using Pearson correlations. An alpha level of 5% (*p* < 0.05) was defined as statistically significant. The statistical analyses were performed using Stata 15.1.

## Results

**Figure 1:**
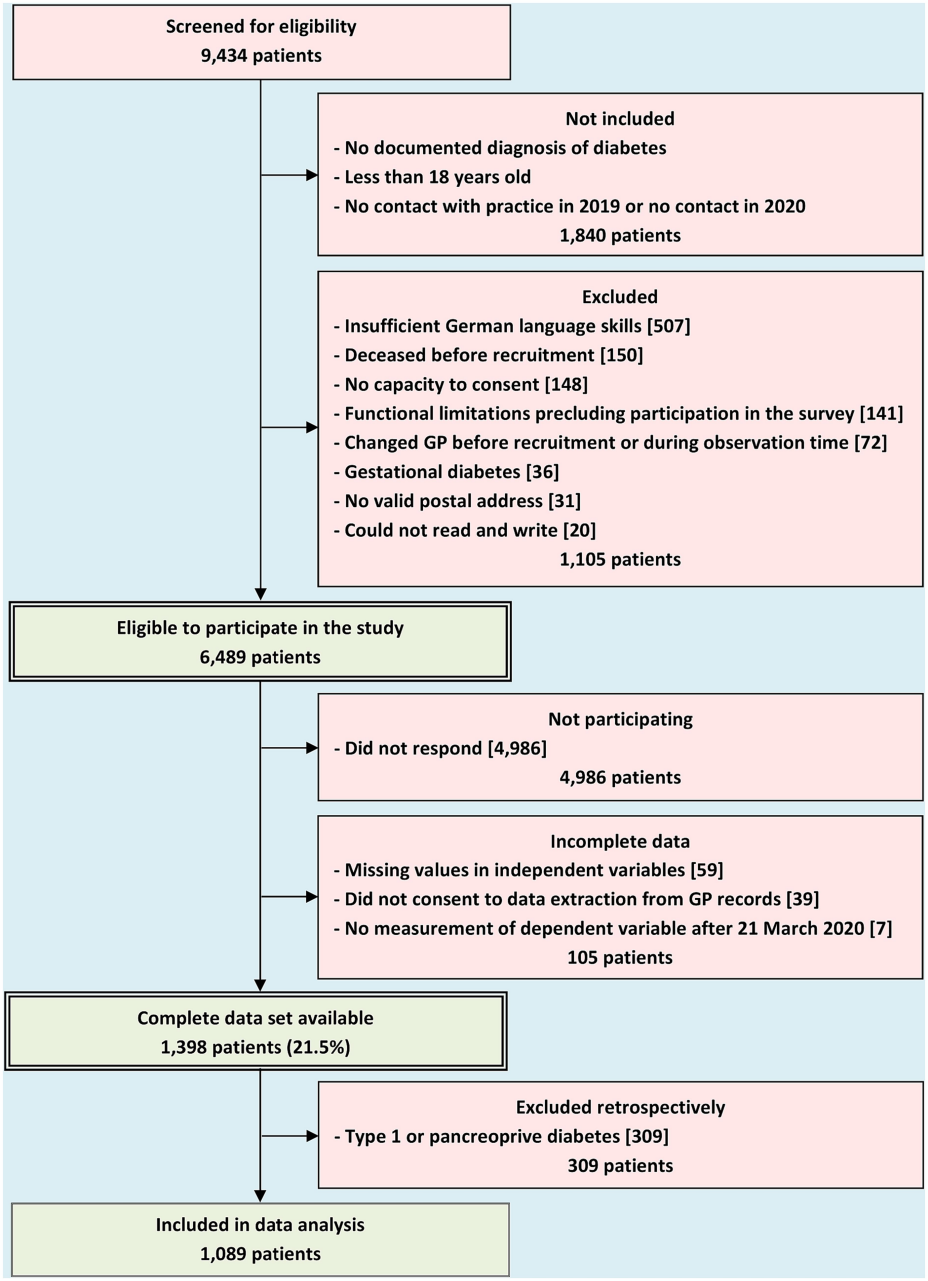
Recruitment of participants.

Patient recruitment is described in Figure 1. Of 9,434 screened patients, 2,945 did not meet criteria for eligibility and 4,986 did not respond. Of 1,503 patients returning a questionnaire, 105 could not be analyzed due to incomplete information. Of 1,398 participating patients with complete data set (21.5% of eligible patients), 309 were excluded retrospectively because of type 1 or pancreoprive diabetes, and 1,089 could be included in our data analysis. All patients had at least two measurements of HbA1c. In addition to the baseline measurement, a median of 4 (interquartile range: 2/6) follow-up measurements of HbA1c per patient were documented during the observation time, which covered the time between 17 January 2019 and 23 December 2021. The total number of HbA1c follow-up observations was 7,987. The number of these observations per patient is shown in Supplementary Figure S1.

The patients had a median age of 68 (60/76) years, 623 (57.2%) were male and 466 (42.8%) female. Marital status of 696 patients (63.9%) was married, 146 (13.4%) were single, 139 (12.8%) widowed and 108 (9.9%) divorced. Tertiary education was reported by 137 patients (12.6%), 483 (44.4%) had secondary education and 469 (43.1%) below. Most patients (1,001; 91.9%) had German and 88 (8.1%) other languages than German as native language. Kernel-density estimation of age is shown in Supplementary Figure S2. Before the first lockdown on 22 March 2020, 864 (79.3%) had cardiovascular risk factors, 539 (49.5%) end organ damages, 217 (19.9%) ASCVD, and 86 (7.9%) cardiovascular events. Heart failure had been diagnosed in 21 (1.9%) patients (*cf.* Table 1). During the observation time, 733 patients (67.3%) received Metformin, 466 (42.8%) intermediate- or long-acting insulins, 410 (37.7%) fast-acting or mixed insulins, 338 (31.0%) sodium-glucose co-transporter 2 (SGLT2) inhibitors, 331 (30.4%) glucagon-like peptide 1 (GLP1) analogues, 177 (16.3%) dipeptidyl peptidase 4 (DPP4) inhibitors, 139 (12.8%) sulfonylureas and 9 (0.8%) Repaglinide (*cf.* Table 2). There were no missing values in the analyzed data.

**Table 1:** Comorbidity before the first lockdown on 22 March 2020 (*n* = 1,089).

Characteristic	Distribution
**Cardiovascular risk factors**, thereof: - Hypertension - Obesity - Dyslipidemia - Tobacco abuse	**864 (79.3%)** 660 (60.6%) 531 (48.8%) 456 (41.9%) 62 (5.7%)
**End organ damages**, thereof: - Neuropathy - Nephropathy - Diabetic foot syndrome - Retinopathy - Maculopathy	**539 (49.5%)** 405 (37.2%) 212 (19.5%) 92 (8.4%) 68 (6.2%) 4 (0.4%)
**Atherosclerotic cardiovascular disease**, thereof: - Chronic ischaemic heart disease - Peripheral arterial occlusive disease - Atherosclerosis	**217 (19.9%)** 194 (17.8%) 39 (3.6%) 5 (0.5%)
**Cardiovascular events**, thereof: - Stroke - Myocardial infarction	**86 (7.9%)** 48 (4.4%) 44 (4.0%)
**Heart failure**	**21 (1.9%)**

*n*: number of participants.

**Table 2:** Diabetes medication prescribed at least once during observation time (*n* = 1,089).

**Characteristic**	**Distribution**
**A10BA Biguanides**, thereof - A10BA02 Metformin	**733 (67.3%)** 733 (67.3%)
**A10AC/A10AE Insulins for injection, intermediate- or long-acting, thereof:** - A10AE04 Insulin glargine, long-acting - A10AE05 Insulin detemir, long-acting - A10AE06 Insulin degludec, long-acting - A10AC01 Insulin (human), intermediate acting	**466 (42.8%)** 309 (28.4%) 97 (8.9%) 58 (5.3%) 50 (4.6%)
**A10AB Insulins for injection, fast-acting or mixed, including** - A10AB05 Insulin aspart, fast acting - A10AB04 Insulin lispro, fast acting - A10AB01 Insulin (human), fast acting - A10AB06 Insulin glulisine, fast acting - A10AD01 Insulin (human), intermediate- or long- combined with fast-acting - A10AD05 Insulin aspart, intermediate- or long- combined with fast-acting	**410 (37.7%)** 145 (13.3%) 121 (11.1%) 116 (10.7%) 55 (5.1%) 41 (3.8%) 4 (0.4%)
**A10BK Sodium-glucose co-transporter 2 (SGLT2) inhibitors**, thereof: - A10BK03 Empagliflozin - A10BK01 Dapagliflozin	**338 (31.0%)** 209 (19.2%) 147 (13.5%)
**A10BJ Glucagon-like peptide 1 (GLP1) analogues**, thereof: - A10BJ05 Dulaglutide - A10BJ06 Semaglutide - A10BJ02 Liraglutide - A10BJ01 Exenatide - A10BJ03 Lixisenatide	**331 (30.4%)** 182 (16.7%) 124 (11.4%) 64 (5.9%) 3 (0.3%) 1 (0.1%)
**A10BH Dipeptidyl peptidase 4 (DPP4) inhibitors**, thereof: - A10BH01 Sitagliptin - A10BH03 Saxagliptin - A10BH05 Linagliptin	**177 (16.3%)** 174 (16.0%) 2 (0.2%) 2 (0.2%)
**A10BB Sulfonylureas**, thereof - A10BB01 Glibenclamide - A10BB12 Glimepiride	**139 (12.8%)** 76 (7.0%) 72 (6.6%)
**A10BX Meglitinides**, thereof - A10BX02 Repaglinide	**9 (0.8%)** 9 (0.8%)

*n*: number of participants.

**Figure 2:**
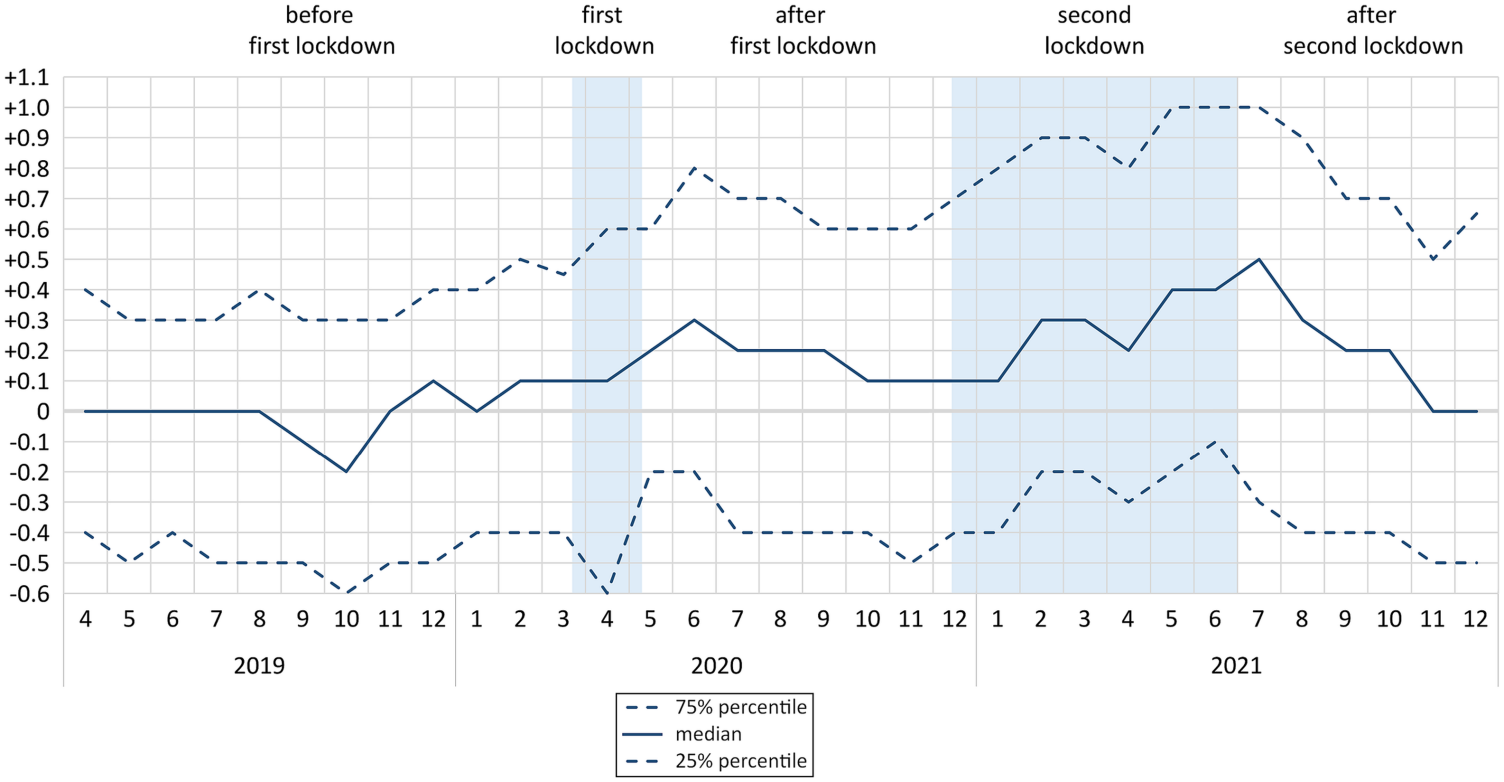
Median and interquartile range of change* in HbA1c levels (in %) over time by phases of COVID-19 pandemic (n = 1,089; *N* = 7,970**) * compared to first measurement; ** 17 observations between January and March 2019 excluded; n: number of participants; N: number of observations.

At first measurement, median HbA1c level (in %) was 6.9 (6.3/7.7). The change in HbA1c levels over time compared to the previous measurement is shown in Figure 2. Sex differences in the HbA1c change over time can be found in Supplementary Figure S3. In this figure, the months January to March 2019 are not represented due to a low number of follow-up observations (*n* = 3 in January 2019, *n* = 4 in February 2020, and *n* = 10 in March 2020). During both lockdowns, HbA1c levels were increasing. In June 2020, after first lockdown, a first peak at +0.3 (−0.2/+0.8) could be observed. In July 2021, after second lockdown, a second peak occurred at +0.5 (−0.3/+1.0). From August 2021 on, these levels were stabilizing and in November 2021 they had reached the baseline of 0 (−0.5/+0.5) again. Kernel-density estimation of HbA1c including all follow-up measurements is shown in Supplementary Figure S4.

Correlation matrix of independent variables is shown in Supplementary Table S1. Excluding correlations between dummies of the same categorical variables, the only association in our data set with |r| ≥ 0.3 was found between age and being widowed (r = 0.33).

Associations between patient characteristics and change in HbA1c levels can be found in Table 3 and a comparison between effect sizes is shown in Supplementary Figure S5. There were significant differences between the phases of the COVID-19 pandemic. As compared to the time before the first lockdown, all subsequent phases were associated with an increase in HbA1c levels, ie, during first lockdown (0.21, 0.11/0.31, *p* < 0.001), after first lockdown (0.23, 0.18/0.28, p < 0.001), during second lockdown (0.40, 0.33/0.47, p < 0.001) and after second lockdown (0.27, 0.18/0.36, p < 0.001). Also, measurement number (−0.03, −0.04/−0.02, p < 0.001) and HbA1c in the previous observation (0.33, 0.32/0.35, p < 0.001) had significant influence on this change.

**Table 3:** Association between patient characteristics and change in HbA1c levels (in %): results from multilevel mixed effects linear regression analysis (n = 1,089; N = 7,987).

Characteristic	ß (95% CI)	*p*
Phase of COVID-19 pandemic: - before first lockdown (1 January 2019 to 21 March 2020) - during first lockdown (22 March 2020 to 6 May 2020) - after first lockdown (7 May 2020 to 12 December 2020) - during second lockdown (13 December 2020 to 30 June 2021) - after second lockdown (1 July 2021 to 31 December 2021)	reference 0.21 (0.11/0.31) 0.23 (0.18/0.28) 0.40 (0.33/0.47) 0.27 (0.18/0.36)	< 0.001 < 0.001 < 0.001 < 0.001
Measurement number	−0.03 (−0.04/−0.02)	<0.001
HbA1c at previous measurement	0.33 (0.32/0.35)	<0.001
Age	−0.001 (−0.004/0.003)	0.640
Sex: - female - male	reference 0.08 (0.01/0.15)	0.019
Marital status: - single - married - widowed - divorced	reference -0.05 (−0.15/0.05) 0.19 (0.05/0.32) 0.04 (−0.09/0.18)	0.297 0.008 0.553
Native language: - German - other than German	reference 0.12 (0.005/0.24)	0.041
Educational level (pursuant to CASMIN): - inadequately completed, general elementary or basic vocational - secondary school certificate or “A” level equivalent - higher or lower tertiary education	reference -0.02 (−0.09/0.05) -0.09 (−0.19/0.01)	0.591 0.087
Comorbidity before first lockdown (22 March 2020): - no ASCVD, no end organ damage, and low cardiovascular risk - no ASCVD, no end organ damage, and high cardiovascular risk - no ASCVD and at least one end organ damage - ASCVD and no cardiovascular event - ASCVD and at least one cardiovascular event - Heart failure	reference 0.10 (−0.01/0.21) 0.12 (0.01/0.23) 0.23 (0.10/0.36) 0.25 (0.10/0.40) -0.10 (−0.34/0.13)	0.090 0.039 0.001 0.001 0.400
Diabetes medication in the 90 days before HbA1c measurement: - no medication - oral medication, but no GLP1 analogues and no insulins - oral medication and GLP1 analogues, but no insulins - intermediate- or long-acting insulins, but no fast-acting or mixed - fast-acting or mixed insulins	reference 0.09 (0.03/0.15) 0.003 (−0.08/0.08) 0.24 (0.16/0.32) 0.30 (0.23/0.36)	0.002 0.938 < 0.001 < 0.001

*n*: number of participants; N: number of observations; CI: confidence interval; CASMIN: Comparative Analysis of Social Mobility in Industrial Nations; ASCVD: atherosclerotic cardiovascular disease; CV: cardiovascular; DPP4: Dipeptidyl peptidase 4; SGLT2: Sodium-glucose co-transporter 2; GLP1: Glucagon-like peptide 1.

In the multivariable models, the increase of HbA1c levels was more pronounced in male patients (0.08, 0.01/0.15, *p* = 0.019), if patients did not have German as native language (0.12, 0.01/0.23, *p* = 0.041), and if they were widowed (0.19 in comparison to being single, 0.05/0.32, *p* = 0.008). End organ damages (0.12, 0.01/0.23, *p* = 0.039), ASCVD (0.23, 0.10/0.36, *p* = 0.001), and cardiovascular events (0.25, 0.10/0.40, p = 0.001) as well as oral medication (0.09, 0.03/0.15, *p* = 0.002), intermediate- or long-acting insulins (0.24, 0.16/0.32, *p* < 0.001), and fast-acting or mixed insulins (0.30, 0.23/0.36, *p* < 0.001) were also related to a greater increase in HbA1c levels (*cf.* Table 3).

Multivariable models comparing the time before and during the pandemic are shown in Supplementary Tables S2, S3. Before the pandemic, increased HbA1c levels were associated with male sex (0.08, 0.01/014, *p* = 0.019), not having German as native language (0.12, 0.01/0.24, *p* = 0.033), ASCVD (0.13, 0.004/0.25, *p* = 0.043) as well as oral medication (0.14, 0.05/0.22, *p* = 0.001), intermediate- or long-acting insulins (0.32, 0.20/0.44, *p* < 0.001) and fast-acting or mixed insulins (0.35, 0.26/0.44, *p* < 0.001). During the pandemic, increased HbA1c levels were associated with being widowed (0.22, 0.08/0.35, *p* = 0.002), ASCVD (0.21, 0.08/0.34, p = 0.001), and cardiovascular events (0.26, 0.12/0.41, *p* < 0.001) as well as oral medication (0.15, 0.08/0.21, *p* < 0.001), intermediate- or long-acting insulins (0.29, 0.19/0.38, *p* < 0.001), and fast-acting or mixed insulins (0.38, 0.31/0.46, *p* < 0.001).

The results of unadjusted analyses of our independent variables can be found in Supplementary Tables S4–S10. In these analyses, increased HbA1c levels were associated with higher age (0.005, 0.002/0.008, *p* = 0.003), male sex (0.08, 0.01/0.15, *p* = 0.035), being widowed (0.25, 0.11/0.39, *p* < 0.001), end organ damages (0.18, 0.07/0.30, *p* = 0.002) and ASCVD (0.32, 0.18/0.46, *p* < 0.001), and cardiovascular events (0.34, 0.18/0.50, *p* < 0.001) as well as oral medication (0.09, 0.03/0.14, *p* = 0.003), intermediate- or long-acting insulins (0.24, 0.16/0.32, *p* < 0.001), and fast-acting or mixed insulins (0.31, 0.25/0.38, *p* < 0.001). Additionally, there was a lower increase of HbA1c levels in patients having tertiary education (−0.12, −0.23/−0.01, *p* = 0.036).

## Discussion

### Statement of principal findings

During the lockdown phases of the COVID-19 pandemic, the median HbA1c levels (in %) increased by up to +0.5. For 25% of the population, this increase was even up to +1.0 or more. As demonstrated in other studies, each 1% increase in HbA1c significantly aggravates the risk of myocardial infarction and mortality (8, 31). The observed increase in HbA1c was more pronounced in patients who are vulnerable due to impaired health status – as indicated by pre-pandemic diagnoses of end organ damages, ASCVD, and cardiovascular events – and intensive diabetes medication. Other patient groups at risk for unfavorable changes in HbA1c were male patients and patients who were widowed or whose native language is not German. Our sensitivity analyses suggest that health status, diabetes medication and marital status are related to deteriorating HbA1c values during the time of pandemic and lockdowns, while sex and native language might predominantly have had an effect on HbA1c in the time before the pandemic.

### Comparison with the literature

In the unadjusted analyses of our study, older age was associated with a greater increase in HbA1c during our observation time. Other studies reported contradictory findings regarding these items (1). In one study, worsening glycemic control occurred more frequently in older T2D patients after lockdown (20), whereas in another study, mean HbA1c increased significantly in participants aged <50 years (18). The results of a meta-analysis revealed no difference between age groups (7). Uni- and multivariate logistic regression analyses of two studies showed no association between sex and glycemic worsening after lockdown (10, 20). In our analysis male patients were at greater risk of more unfavorable changes in HbA1c, but this effect was predominantly related to the time before COVID-19.

Prior studies have rarely examined the effect of education on HbA1c levels during lockdown. However, D’Onofrio et al. found no differences between HbA1c levels before and after lockdown depending on education (19), whereas our unadjusted analyses indicated that patients with tertiary education had a lower risk of adverse HbA1c changes. In general, T2D patients with high education have lower HbA1c levels (26). Previous findings suggest an association between low educational attainment and behaviors that increase the risk of blood glucose deterioration, such as an inactive lifestyle and poor dietary habits (32, 33).

In Europe, there are many ethnic minorities that have a higher prevalence of T2D than their native counterparts. For example, people from the Middle East and North Africa living in Europe have a two to four times higher risk of T2D than their European host population (34). In city of Hamburg, six hundred and fifty thousand people have a migration background, which is 35% of the total population (35). However, we observed that T2D patients who did not have German as native language were at greater risk of more unfavorable changes in HbA1c, which could be due to lower health literacy among immigrants compared with natives (32, 36, 37). In addition to the language barrier, people with immigrant backgrounds often have lower SES than natives, which can affect access to care, communication with health care providers, treatment decisions, and ability to adhere to recommended therapy (26, 27). Patients with low SES have been shown to have higher HbA1c than those with high SES (26), and there is a clear association between low SES and diabetes complications, particularly retinopathy and cardiopathy (38). In our data, the effect of native language was mostly seen in the time before the pandemic.

We also observed that widowed T2D patients had a higher risk of more unfavorable changes in HbA1c levels, which could be due to a lack of social support (28).

In our study population, we found that ASCVD, previous cardiovascular events and insulin treatment were associated with a greater increase in HbA1c. High HbA1c is significantly associated with retinopathy (39) as well as cardiac events and increased all-cause mortality (40). And, the risk of all-cause mortality is higher in patients treated with insulin than in those receiving oral combination medications (31, 40). A previous T2D study performed by Biamonte et al. found that insulin therapy was the only significant independent predictor of HbA1c worsening during the first lockdown (10). Falcetta et al. also observed that HbA1c worsening occurred more frequently in individuals on insulin therapy (20). In T2D, intensified treatment such as insulin therapy is often used in patients with reduced functional beta-cell activity that occurs with long-term disease. In addition, insulin is preferred for comorbidities such as chronic kidney disease, which can be a contraindication to oral antidiabetic drugs (10, 20). Our results suggest that the glycemic control of patients on insulin therapy tends to be unstable and therefore is more likely to deteriorate in crisis situations.

### Implications for clinical practice

Elevated HbA1c has been shown to be associated with micro- and macrovascular comorbidities, cardiac events, and increased total mortality (8, 31, 39, 40). Thus, it is important to prevent deterioration of glycemic control in diabetic patients, even in crisis.

Our results clearly demonstrated that glycemic control of T2D patients may deteriorate during stressful situations such as pandemic lockdowns. We identified subgroups that are at higher risk of glycemic worsening and therefore require special medical attention. In particular, patients with ASCVD, cardiovascular events, and those on insulin therapy appear to be at highest risk for worsening glycemic control. Lack of social support (e.g., patients living alone after the death of their spouse) or low SES (e.g., low education or due to migration background) are other risk factors that may lead to worsening glycemic control due to contact limitations and health care restrictions.

In contrast to T2D, T1D patients use digital treatments such as continuous glucose monitoring (CGM), flash glucose monitoring (FGM) and hybrid closed loop system (HCL) more often, which had a positive impact on glycemic control during lockdown (41–43). These findings suggest that T2D patients might also benefit from digital diabetes management in stressful situations as FGM and CGM might also help T2D patients keep their blood glucose levels stable in future pandemics (9). In addition, telemedicine could help overcome physical barriers and improve access to healthcare (not only) in a pandemic context (3).

### Strengths and limitations

Compared with other studies analyzing the association between COVID-19 lockdown and glycemic control, CoDiaM has a long observation time of 36 months, a large sample size of 1,089 patients, and it is based on multilevel, multivariable analyses, which allow for cluster effects on the level of healthcare providers and include important covariates like marital status, education, native language, comorbidities and diabetes medication.

Other strengths of our study include high data quality of the dependent variable (ie, laboratory results from blood tests), a complete data set without missing values, and a median of four follow-up measurements per patient of the outcome variable. The robustness of the results is confirmed by sensitivity analyses comparing the time before and during the pandemic and by additional analyses examining the unadjusted effect of the independent variables on the outcome.

A limitation of the study is the low participation rate of 21.5%, which could be related to a higher rate of better educated and more healthy living patients participating in our study. Therefore, changes during lockdowns and association with other factors could have been estimated too conservatively. However, despite possible effects on the representativeness of samples, low participation rates usually do not affect the association identified in the data set (44–47). Also, low participation rates are common to many COVID-19 studies (eg, 12% in Ayoubkhani et al. 2022 (48)). Other factors possibly affecting representativeness of our study are a low number of included health care providers (n = 14) and limiting the study area to the city of Hamburg, ie, one metropolitan area in Germany. Patients who died during the COVID-19 pandemic are not represented in our data set. As worse glycemic control is related to an increased risk of death due to COVID-19 (5), effects of pandemic and lockdown on HbA1c levels might have been underestimated.

It needs to be noted that only patients with T2D were considered in our analyses, although the CoDiaM study included both T1D and T2D. As T1D differs from T2D in terms of pathogenesis, treatment options and mean age of patients (9), the impact of the Covid-19 pandemic on T1D will be assessed in a separate manuscript.

Additionally, socio-demographic factors only have been assessed at one time during the pandemic and some of them could therefore have changed during observation time without our knowledge, particularly marital status. However, the frequency of changes within these indicators is usually not very high. It is also possible that factors such as depressive disorders, which are not represented in our statistical models, had an additional effect on HbA1c values during the observation time.

## Conclusion

Both lockdowns resulted in an increase in HbA1c levels, which puts T2D patients at higher risk for myocardial infarction and mortality. In particular, patients with ASCVD, previous cardiovascular events, and those on insulin therapy appear to be at highest risk for worsening glycemic control in crisis and thus require special medical attention. Digital solutions such as FGM and CGM could help keep their blood glucose levels stable in future pandemics. In addition, telemedicine might help overcome physical barriers and improve access to healthcare.

## Data availability statement

The raw data supporting the conclusions of this article will be made available by the authors, without undue reservation.

## Ethics statement

The studies involving humans were approved by Local Psychological Ethics Committee at the Center for Psychosocial Medicine of the University Medical Center Hamburg-Eppendorf. The studies were conducted in accordance with the local legislation and institutional requirements. The participants provided their written informed consent to participate in this study.

## Author contributions

IS: Conceptualization, Data curation, Formal analysis, Methodology, Visualization, Writing – original draft, Writing – review & editing. DT: Conceptualization, Methodology, Project administration, Supervision, Writing – original draft, Writing – review & editing. LW: Investigation, Writing – review & editing. LB: Investigation, Writing – review & editing. DL: Conceptualization, Writing – review & editing. MS: Conceptualization, Funding acquisition, Resources, Writing – review & editing.
