# Supplementary material for: Impact of two COVID-19 lockdowns on HbA1c levels in patients with type 2 diabetes and associations with patient characteristics: a multicentre, observational cohort study over three years
Source: Front Public Health. 2024 Jan 5;11:1272769. doi: 10.3389/fpubh.2023.1272769 (PMC10796585; doi:10.3389/fpubh.2023.1272769)
Supplement: Supplementary file 1 [file Data_Sheet_1.docx]

**Impact of two COVID-19 lockdowns on HbA1c levels in patients with type 2 diabetes and associations with patient characteristics.
A multicentre, observational cohort study over three years**

**Supplement**

Ingmar Schäfer, Daniel Tajdar, Laura Walther, Lasse Bittner, Dagmar Lühmann, Martin Scherer

**Figure S1: Number of follow-up observations per patient (n=1,089; N=7,987)**

**
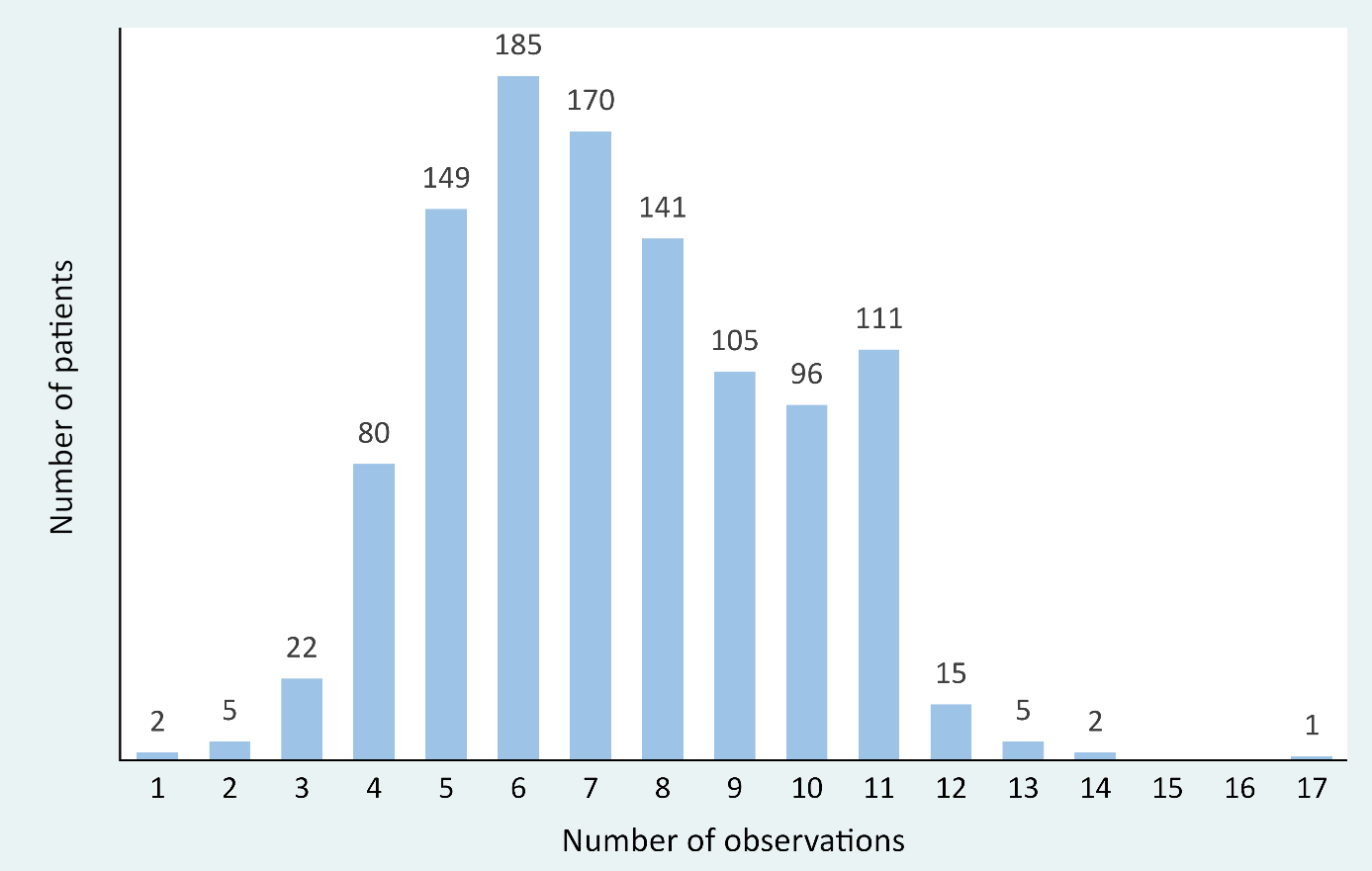
**

**n: number of participants; N: number of observations**

**Figure S2: Kernel density estimation of age (n=1,089)**

**
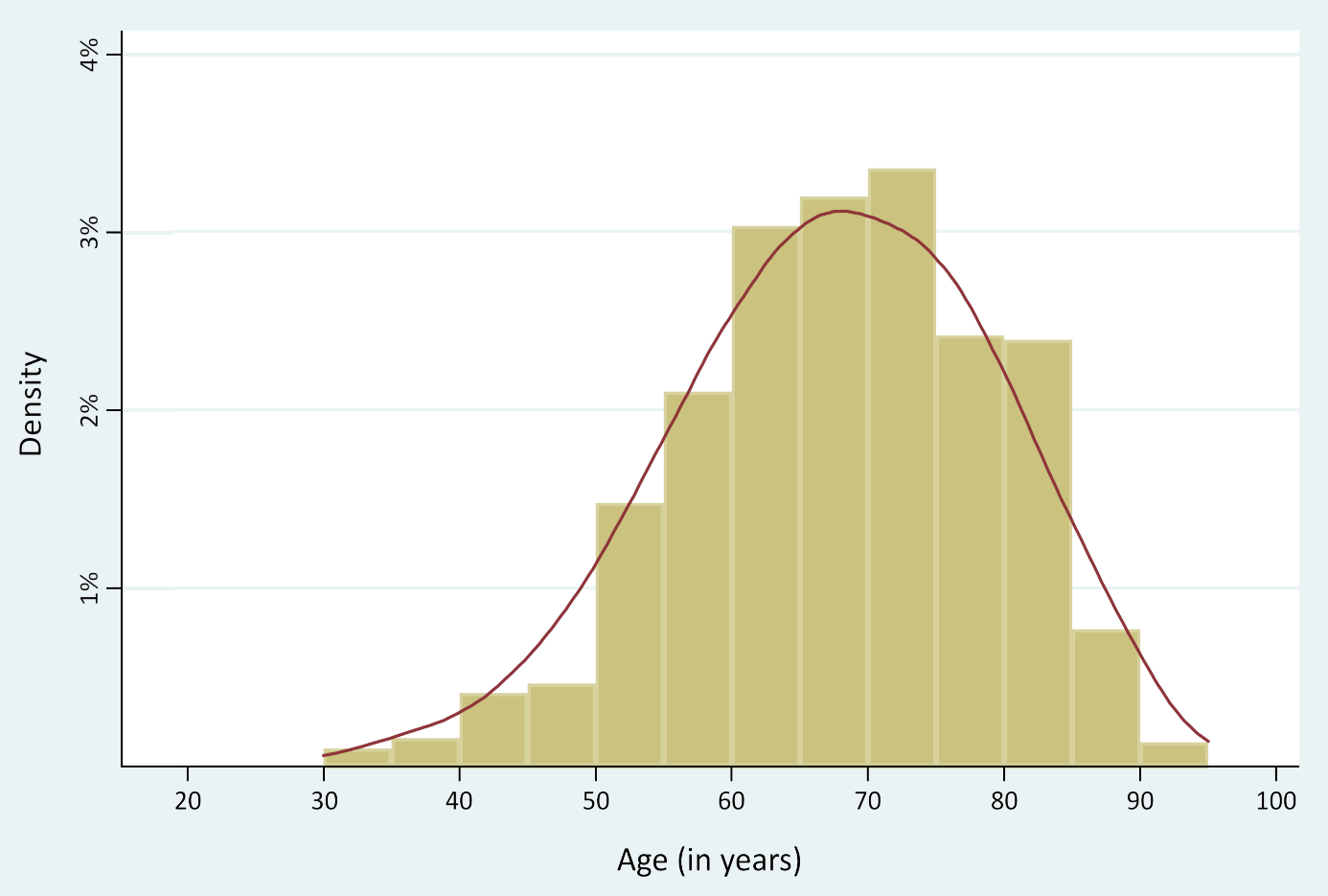
**

**n: number of participants**

**Figure S3: Sex differences in median and interquartile range of change* in HbA1c levels (in %) over time by phases of COVID-19 pandemic (n=1,089; N=7,970**)**

**
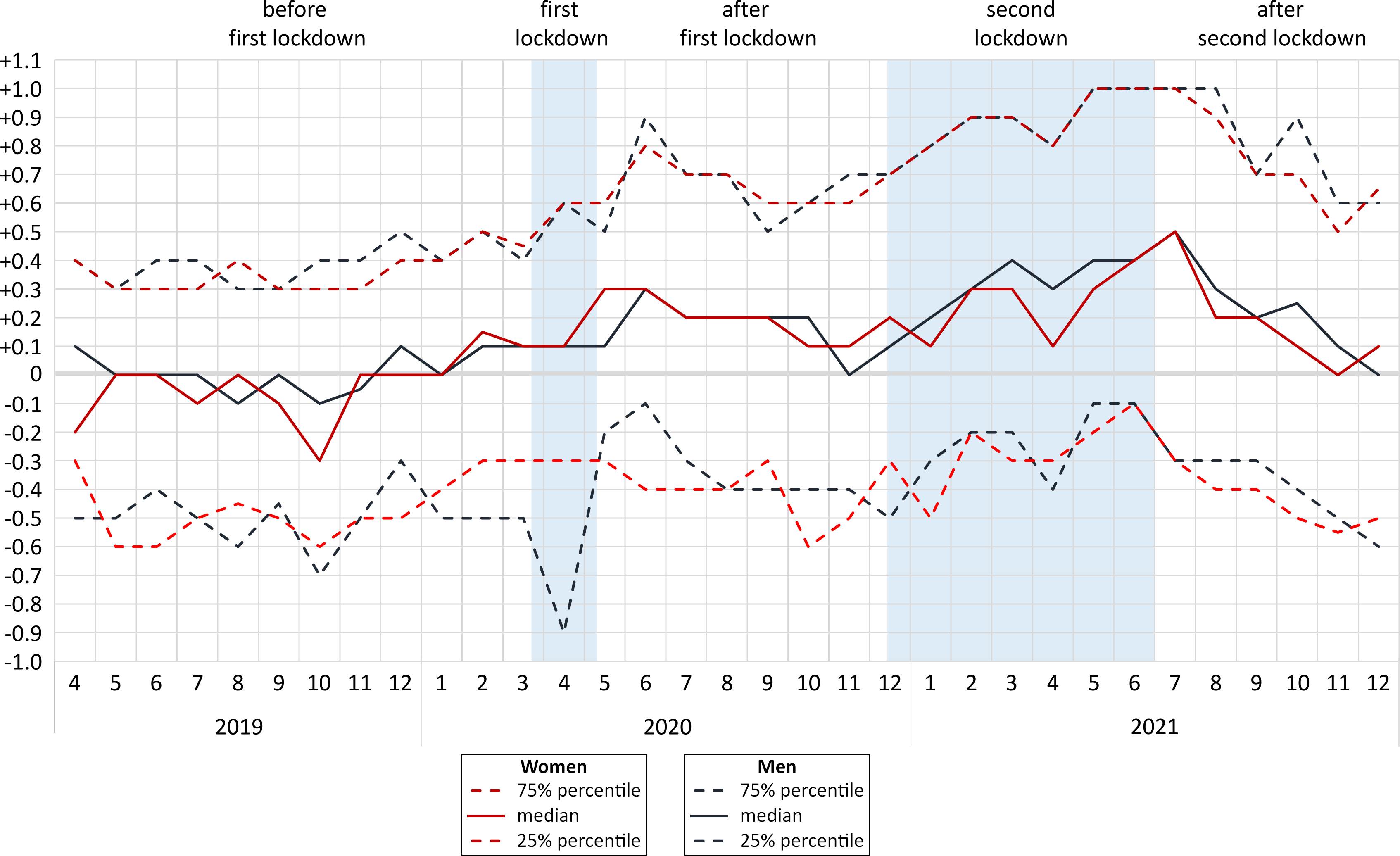
**

* compared to first measurement; ** 17 observations between January and March 2019 excluded; n: number of participants; N: number of observations.

**Figure S4: Kernel density estimation of HbA1c levels (in %; n=1,089; N=7,987)**

**
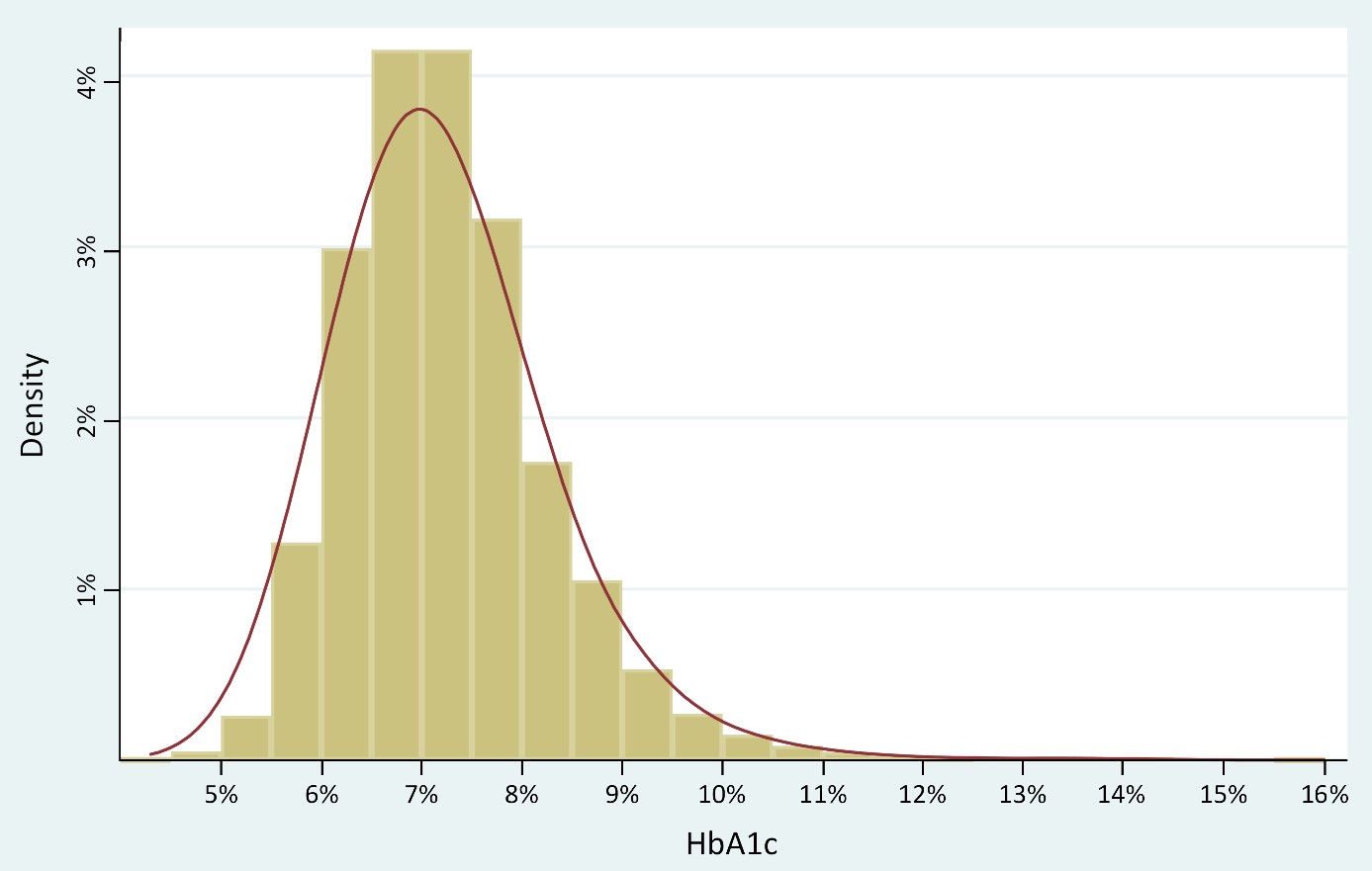
**

**n: number of participants; N: number of observations**

**Figure S5: Comparison between effect sizes of independent variables in main analysis (in %; n=1,089; N=7,987)**


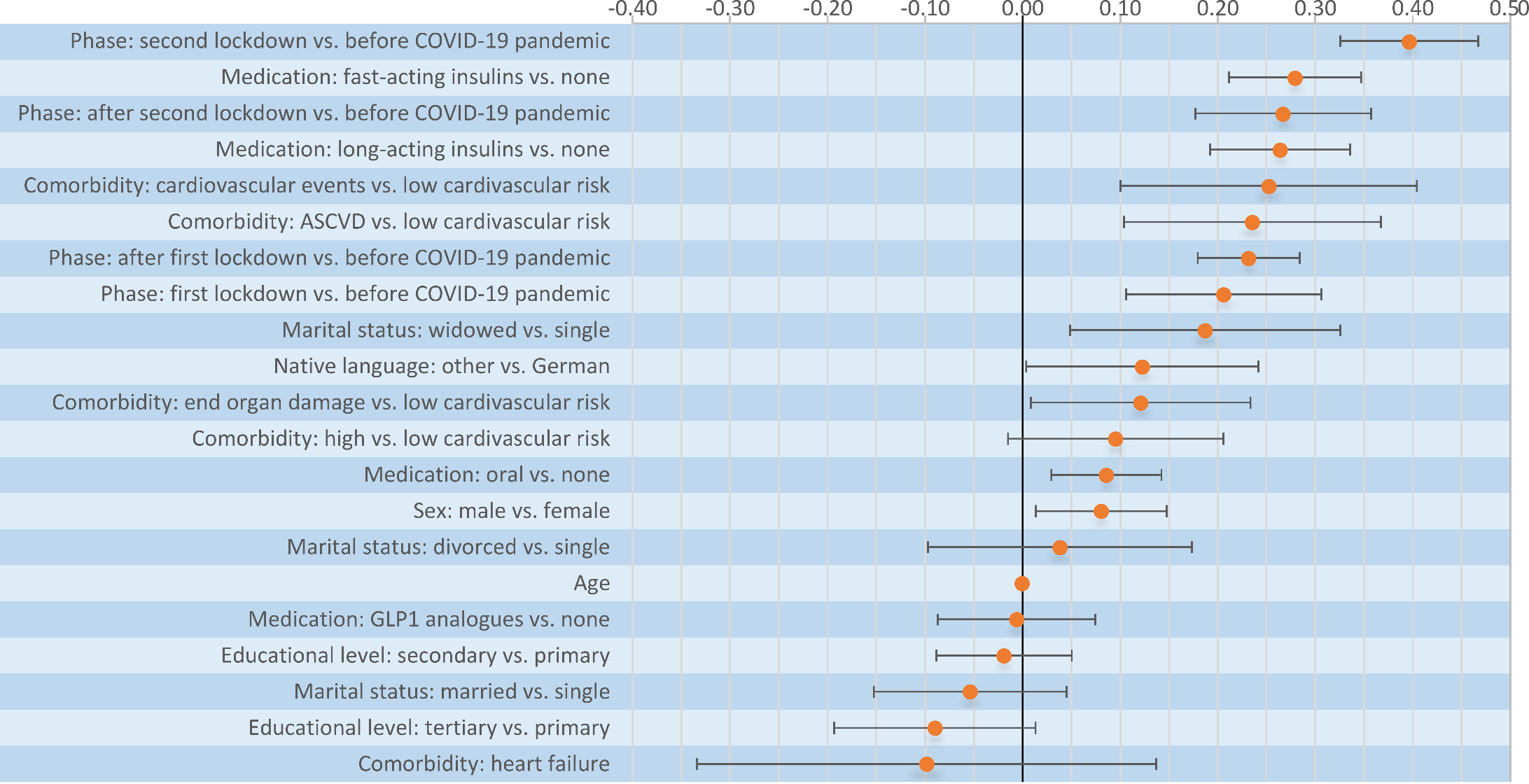


**ASCVD: atherosclerotic cardiovascular disease; GLP1: Glucagon-like peptide 1**

**Table S1: Pearson correlations between independent variables (n=1,089; N=7,987)**

|  | **HbA1c (current)** | **HbA1c (previous)** | **Measurement** | **Phase (1)** | **Phase (2)** | **Phase (3)** | **Phase (4)** | **Age** | **Sex** | **CASMIN3 (1)** | **CASMIN3 (2)** | **Native language** | **Marital status (1)** | **Marital status (2)** | **Marital status (3)** | **Comorbidity (1)** | **Comorbidity (2)** | **Comorbidity (3)** | **Comorbidity (4)** | **Heart failure** | **Medication (1)** | **Medication (2)** | **Medication (3)** | **Medication (4)** |
| --- | --- | --- | --- | --- | --- | --- | --- | --- | --- | --- | --- | --- | --- | --- | --- | --- | --- | --- | --- | --- | --- | --- | --- | --- |
| **HbA1c (current)** | 1.00 |  |  |  |  |  |  |  |  |  |  |  |  |  |  |  |  |  |  |  |  |  |  |  |
| **HbA1c (previous)** | 0.69 | 1.00 |  |  |  |  |  |  |  |  |  |  |  |  |  |  |  |  |  |  |  |  |  |  |
| **Measurement** | 0.06 | 0.07 | 1.00 |  |  |  |  |  |  |  |  |  |  |  |  |  |  |  |  |  |  |  |  |  |
| **Phase (1)** | -0.01 | -0.01 | -0.05 | 1.00 |  |  |  |  |  |  |  |  |  |  |  |  |  |  |  |  |  |  |  |  |
| **Phase (2)** | 0.00 | -0.04 | -0.04 | -0.09 | 1.00 |  |  |  |  |  |  |  |  |  |  |  |  |  |  |  |  |  |  |  |
| **Phase (3)** | 0.09 | 0.04 | 0.29 | -0.08 | -0.29 | 1.00 |  |  |  |  |  |  |  |  |  |  |  |  |  |  |  |  |  |  |
| **Phase (4)** | 0.01 | 0.08 | 0.56 | -0.08 | -0.27 | -0.26 | 1.00 |  |  |  |  |  |  |  |  |  |  |  |  |  |  |  |  |  |
| **Age** | 0.08 | 0.06 | -0.04 | -0.01 | -0.01 | 0.00 | 0.00 | 1.00 |  |  |  |  |  |  |  |  |  |  |  |  |  |  |  |  |
| **Sex** | -0.05 | -0.05 | -0.02 | -0.02 | 0.00 | 0.00 | 0.01 | -0.04 | 1.00 |  |  |  |  |  |  |  |  |  |  |  |  |  |  |  |
| **CASMIN3 (1)** | -0.01 | 0.00 | -0.02 | 0.00 | 0.01 | 0.00 | 0.01 | -0.16 | 0.04 | 1.00 |  |  |  |  |  |  |  |  |  |  |  |  |  |  |
| **CASMIN3 (2)** | -0.05 | -0.05 | 0.01 | 0.02 | 0.00 | -0.01 | -0.01 | -0.04 | -0.07 | -0.34 | 1.00 |  |  |  |  |  |  |  |  |  |  |  |  |  |
| **Native language** | 0.04 | 0.04 | 0.01 | 0.01 | -0.01 | 0.01 | 0.00 | -0.14 | 0.01 | -0.04 | 0.00 | 1.00 |  |  |  |  |  |  |  |  |  |  |  |  |
| **Marital status (1)** | -0.08 | -0.08 | -0.02 | -0.01 | 0.00 | 0.00 | 0.00 | -0.01 | -0.14 | -0.04 | 0.01 | 0.04 | 1.00 |  |  |  |  |  |  |  |  |  |  |  |
| **Marital status (2)** | 0.11 | 0.10 | -0.04 | -0.02 | 0.00 | 0.01 | 0.00 | 0.33 | 0.16 | -0.04 | -0.06 | -0.07 | -0.48 | 1.00 |  |  |  |  |  |  |  |  |  |  |
| **Marital status (3)** | 0.03 | 0.04 | 0.01 | 0.02 | 0.00 | 0.00 | 0.00 | 0.01 | 0.08 | 0.05 | -0.04 | 0.02 | -0.44 | -0.13 | 1.00 |  |  |  |  |  |  |  |  |  |
| **Comorbidity (1)** | -0.05 | -0.05 | -0.05 | -0.01 | 0.01 | 0.00 | 0.01 | -0.25 | 0.09 | 0.08 | 0.01 | 0.03 | 0.02 | -0.06 | -0.09 | 1.00 |  |  |  |  |  |  |  |  |
| **Comorbidity (2)** | 0.01 | 0.01 | 0.00 | -0.01 | -0.01 | 0.01 | -0.01 | 0.21 | 0.06 | -0.04 | -0.02 | -0.08 | -0.04 | 0.08 | 0.05 | -0.46 | 1.00 |  |  |  |  |  |  |  |
| **Comorbidity (3)** | 0.09 | 0.08 | 0.00 | 0.01 | 0.00 | 0.00 | 0.00 | 0.20 | -0.15 | -0.04 | -0.03 | 0.01 | 0.02 | 0.01 | 0.07 | -0.26 | -0.30 | 1.00 |  |  |  |  |  |  |
| **Comorbidity (4)** | 0.07 | 0.07 | 0.01 | -0.01 | 0.00 | -0.01 | -0.01 | 0.12 | -0.05 | -0.03 | 0.00 | -0.01 | -0.01 | 0.03 | 0.00 | -0.18 | -0.22 | -0.12 | 1.00 |  |  |  |  |  |
| **Heart failure** | 0.00 | 0.00 | -0.01 | 0.00 | 0.00 | -0.01 | 0.01 | 0.10 | 0.00 | -0.02 | -0.04 | -0.04 | -0.02 | 0.03 | 0.01 | -0.04 | 0.00 | 0.04 | 0.08 | 1.00 |  |  |  |  |
| **Medication (1)** | -0.10 | -0.12 | -0.07 | 0.02 | 0.01 | 0.00 | -0.03 | -0.04 | -0.04 | 0.01 | 0.01 | 0.03 | 0.05 | -0.05 | -0.02 | 0.07 | -0.06 | -0.01 | -0.04 | -0.05 | 1.00 |  |  |  |
| **Medication (2)** | -0.04 | -0.01 | 0.08 | -0.01 | -0.01 | 0.02 | 0.06 | -0.15 | 0.06 | 0.01 | 0.03 | 0.00 | 0.01 | -0.11 | 0.07 | 0.07 | -0.05 | -0.04 | -0.03 | -0.03 | -0.22 | 1.00 |  |  |
| **Medication (3)** | 0.11 | 0.13 | 0.04 | -0.01 | -0.01 | 0.01 | 0.00 | -0.02 | -0.03 | -0.03 | 0.04 | 0.04 | -0.01 | -0.02 | 0.04 | -0.03 | 0.03 | 0.03 | -0.02 | 0.01 | -0.21 | -0.11 | 1.00 |  |
| **Medication (4)** | 0.28 | 0.27 | 0.01 | -0.02 | -0.01 | 0.02 | 0.00 | 0.16 | 0.00 | 0.00 | -0.07 | -0.07 | -0.02 | 0.15 | -0.05 | -0.12 | 0.11 | 0.06 | 0.09 | 0.03 | -0.42 | -0.23 | -0.21 | 1.00 |

**Table S2: Association between patient characteristics and change in HbA1c levels (in %) before the COVID-19 pandemic: results from multilevel mixed effects linear regression analysis (n=1,057; N=2,615)**

| **Characteristic** | **ß (95% CI)** | **p** |
| --- | --- | --- |
| Measurement number | 0.07 (0.04/0.10) | <0.001 |
| HbA1c at previous measurement | 0.56 (0.54/0.59) | <0.001 |
| Age | 0.0002 (-0.003/0.004) | 0.881 |
| Sex: - female - male | reference 0.08 (0.01/0.14) | 0.019 |
| Marital status: - single - married - widowed - divorced | reference -0.05 (-0.15/0.04) 0.07 (-0.05/0.20) 0.01 (-0.12/0.14) | 0.237 0.257 0.858 |
| Native language: - German - other than German | reference 0.12 (0.01/0.24) | 0.033 |
| Educational level (pursuant to CASMIN):  - inadequately completed, general elementary or basic vocational  - secondary school certificate or “A” level equivalent - higher or lower tertiary education | reference -0.03 (-0.10/0.03) -0.09 (-0.19/0.004) | 0.328 0.062 |
| Comorbidity before first lockdown (22 March 2020): - no ASCVD, no end organ damage, and low cardiovascular risk - no ASCVD, no end organ damage, and high cardiovascular risk - no ASCVD and at least one end organ damage - ASCVD and no cardiovascular event - ASCVD and at least one cardiovascular event  - Heart failure | reference 0.07 (-0.03/0.18) 0.08 (-0.02/0.18) 0.13 (0.004/0.25) 0.07 (-0.07/0.21)  0.08 (-0.13/0.30) | 0.188 0.131 0.043 0.340  0.456 |
| Diabetes medication in the 90 days before HbA1c measurement: - no medication - oral medication, but no GLP1 analogues and no insulins - oral medication and GLP1 analogues, but no insulins - intermediate- or long-acting insulins, but no fast-acting or mixed - fast-acting or mixed insulins | reference 0.14 (0.05/0.22) 0.11 (-0.01/0.24) 0.32 (0.20/0.44) 0.35 (0.26/0.44) | 0.001 0.073 <0.001 <0.001 |

**n: number of participants; N: number of observations; CI: confidence interval;
CASMIN: Comparative Analysis of Social Mobility in Industrial Nations; ASCVD: atherosclerotic cardiovascular disease (ASCVD); CV: cardiovascular; DPP4: Dipeptidyl peptidase 4; SGLT2: Sodium-glucose co-transporter 2; GLP1: Glucagon-like peptide 1**

**Table S3: Association between patient characteristics and change in HbA1c levels (in %) during the COVID-19 pandemic: results from multilevel mixed effects linear regression analysis (n=1,089; N=5,372)**

| **Characteristic** | **ß (95% CI)** | **p** |
| --- | --- | --- |
| Measurement number | -0.02 (-0.03/-0.01) | <0.001 |
| HbA1c at previous measurement | 0.40 (0.37/0.42) | <0.001 |
| Age | -0.001 (-0.005/0.002) | 0.423 |
| Sex: - female - male | reference 0.06 (-0.01/0.12) | 0.086 |
| Marital status: - single - married - widowed - divorced | reference -0.01 (-0.11/0.08) 0.22 (0.08/0.35) 0.05 (-0.08/0.18) | 0.788 0.002 0.443 |
| Native language: - German - other than German | reference 0.10 (-0.02/0.21) | 0.105 |
| Educational level (pursuant to CASMIN):  - inadequately completed, general elementary or basic vocational  - secondary school certificate or “A” level equivalent - higher or lower tertiary education | reference -0.01 (-0.08/0.06) -0.06 (-0.16/0.04) | 0.799 0.224 |
| Comorbidity before first lockdown (22 March 2020): - no ASCVD, no end organ damage, and low cardiovascular risk - no ASCVD, no end organ damage, and high cardiovascular risk - no ASCVD and at least one end organ damage - ASCVD and no cardiovascular event - ASCVD and at least one cardiovascular event  - Heart failure | reference 0.09 (-0.01/0.20) 0.09 (-0.02/0.20) 0.21 (0.08/0.34) 0.26 (0.12/0.41)  -0.13 (-0.36/0.10) | 0.082 0.100 0.001 <0.001  0.275 |
| Diabetes medication in the 90 days before HbA1c measurement: - no medication - oral medication, but no GLP1 analogues and no insulins - oral medication and GLP1 analogues, but no insulins - intermediate- or long-acting insulins, but no fast-acting or mixed - fast-acting or mixed insulins | reference 0.15 (0.08/0.21) 0.07 (-0.03/0.16) 0.29 (0.19/0.38) 0.38 (0.31/0.46) | <0.001 0.167 <0.001 <0.001 |

**n: number of participants; N: number of observations; CI: confidence interval;
CASMIN: Comparative Analysis of Social Mobility in Industrial Nations; ASCVD: atherosclerotic cardiovascular disease (ASCVD); CV: cardiovascular; DPP4: Dipeptidyl peptidase 4; SGLT2: Sodium-glucose co-transporter 2; GLP1: Glucagon-like peptide 1**

**Table S4: Association between age and change in HbA1c levels (in %):
results from multilevel mixed effects linear regression analysis (n=1,089; N=7,987)**

| **Characteristic** | **ß (95% CI)** | **p** |
| --- | --- | --- |
| Phase of COVID-19 pandemic: - before first lockdown (1 January 2019 to 21 March 2020) - during first lockdown (22 March 2020 to 6 May 2020) - after first lockdown (7 May 2020 to 12 December 2020) - during second lockdown (13 December 2020 to 30 June 2021) - after second lockdown (1 July 2021 to 31 December 2021) | reference 0.20 (0.10/0.30) 0.23 (0.18/0.28) 0.40 (0.33/0.47) 0.27 (0.18/0.36) | <0.001 <0.001 <0.001 <0.001 |
| Measurement number | -0.03 (-0.04/-0.02) | <0.001 |
| HbA1c at previous measurement | 0.33 (0.31/0.35) | <0.001 |
| Age | 0.005 (0.002/0.008) | 0.003 |

**n: number of participants; N: number of observations; CI: confidence interval**

**Table S5: Association between sex and change in HbA1c levels (in %):
results from multilevel mixed effects linear regression analysis (n=1,089; N=7,987)**

| **Characteristic** | **ß (95% CI)** | **P** |
| --- | --- | --- |
| Phase of COVID-19 pandemic: - before first lockdown (1 January 2019 to 21 March 2020) - during first lockdown (22 March 2020 to 6 May 2020) - after first lockdown (7 May 2020 to 12 December 2020) - during second lockdown (13 December 2020 to 30 June 2021) - after second lockdown (1 July 2021 to 31 December 2021) | reference 0.20 (0.10/0.30) 0.23 (0.18/0.28) 0.40 (0.33/0.47) 0.27 (0.18/0.36) | <0.001 <0.001 <0.001 <0.001 |
| Measurement number | -0.03 (-0.04/-0.02) | <0.001 |
| HbA1c at previous measurement | 0.33 (0.31/0.35) | <0.001 |
| Sex: - female - male | reference 0.08 (0.01/0.15) | 0.035 |

**n: number of participants; N: number of observations; CI: confidence interval**

**Table S6: Association between marital status and change in HbA1c levels (in %):
results from multilevel mixed effects linear regression analysis (n=1,089; N=7,987)**

| **Characteristic** | **ß (95% CI)** | **P** |
| --- | --- | --- |
| Phase of COVID-19 pandemic: - before first lockdown (1 January 2019 to 21 March 2020) - during first lockdown (22 March 2020 to 6 May 2020) - after first lockdown (7 May 2020 to 12 December 2020) - during second lockdown (13 December 2020 to 30 June 2021) - after second lockdown (1 July 2021 to 31 December 2021) | reference 0.20 (0.10/0.30) 0.23 (0.18/0.28) 0.40 (0.32/0.47) 0.26 (0.17/0.35) | <0.001 <0.001 <0.001 <0.001 |
| Measurement number | -0.03 (-0.04/-0.02) | <0.001 |
| HbA1c at previous measurement | 0.33 (0.31/0.35) | <0.001 |
| Marital status: - single - married - widowed - divorced | reference -0.02 (-0.12/0.09) 0.25 (0.11/0.39) 0.07 (-0.08/0.21) | 0.714 <0.001 0.376 |

**n: number of participants; N: number of observations; CI: confidence interval**

**Table S7: Association between native language and change in HbA1c levels (in %):
results from multilevel mixed effects linear regression analysis (n=1,089; N=7,987)**

| **Characteristic** | **ß (95% CI)** | **P** |
| --- | --- | --- |
| Phase of COVID-19 pandemic: - before first lockdown (1 January 2019 to 21 March 2020) - during first lockdown (22 March 2020 to 6 May 2020) - after first lockdown (7 May 2020 to 12 December 2020) - during second lockdown (13 December 2020 to 30 June 2021) - after second lockdown (1 July 2021 to 31 December 2021) | reference 0.20 (0.10/0.30) 0.23 (0.18/0.28) 0.40 (0.33/0.47) 0.26 (0.17/0.36) | <0.001 <0.001 <0.001 <0.001 |
| Measurement number | -0.03 (-0.04/-0.02) | <0.001 |
| HbA1c at previous measurement | 0.33 (0.31/0.35) | <0.001 |
| Native language: - German - other than German | reference 0.09 (-0.04/0.22) | 0.160 |

**n: number of participants; N: number of observations; CI: confidence interval**

**Table S8: Association between educational level and change in HbA1c levels (in %):
results from multilevel mixed effects linear regression analysis (n=1,089; N=7,987)**

| **Characteristic** | **ß (95% CI)** | **P** |
| --- | --- | --- |
| Phase of COVID-19 pandemic: - before first lockdown (1 January 2019 to 21 March 2020) - during first lockdown (22 March 2020 to 6 May 2020) - after first lockdown (7 May 2020 to 12 December 2020) - during second lockdown (13 December 2020 to 30 June 2021) - after second lockdown (1 July 2021 to 31 December 2021) | reference 0.20 (0.10/0.30) 0.23 (0.18/0.28) 0.40 (0.33/0.47) 0.26 (0.17/0.36) | <0.001 <0.001 <0.001 <0.001 |
| Measurement number | -0.03 (-0.04/-0.02) | <0.001 |
| HbA1c at previous measurement | 0.33 (0.31/0.35) | <0.001 |
| Educational level (pursuant to CASMIN):  - inadequately completed, general elementary or basic vocational  - secondary school certificate or “A” level equivalent - higher or lower tertiary education | reference -0.04 (-0.12/0.03) -0.12 (-0.23/-0.01) | 0.255 0.036 |

**n: number of participants; N: number of observations; CI: confidence interval;
CASMIN: Comparative Analysis of Social Mobility in Industrial Nations**

**Table S9: Association between comorbidity before first lockdown and change in HbA1c levels
(in %): results from multilevel mixed effects linear regression analysis (n=1,089; N=7,987)**

| **Characteristic** | **ß (95% CI)** | **P** |
| --- | --- | --- |
| Phase of COVID-19 pandemic: - before first lockdown (1 January 2019 to 21 March 2020) - during first lockdown (22 March 2020 to 6 May 2020) - after first lockdown (7 May 2020 to 12 December 2020) - during second lockdown (13 December 2020 to 30 June 2021) - after second lockdown (1 July 2021 to 31 December 2021) | reference 0.21 (0.11/0.31) 0.23 (0.18/0.28) 0.41 (0.34/0.48) 0.27 (0.18/0.36) | <0.001 <0.001 <0.001 <0.001 |
| Measurement number | -0.03 (-0.05/-0.02) | <0.001 |
| HbA1c at previous measurement | 0.33 (0.31/0.35) | <0.001 |
| Comorbidity before first lockdown (22 March 2020): - no ASCVD, no end organ damage, and low cardiovascular risk - no ASCVD, no end organ damage, and high cardiovascular risk - no ASCVD and at least one end organ damage - ASCVD and no cardiovascular event - ASCVD and at least one cardiovascular event  - Heart failure | reference 0.10 (-0.02/0.22) 0.18 (0.07/0.30) 0.32 (0.18/0.46) 0.34 (0.18/0.50)  -0.10 (-0.36/0.16) | 0.096 0.002 <0.001 <0.001  0.447 |

**n: number of participants; N: number of observations; CI: confidence interval**

**Table S10: Association between intensity of diabetes medication and change in HbA1c levels (in %):
results from multilevel mixed effects linear regression analysis (n=1,089; N=7,987)**

| **Characteristic** | **ß (95% CI)** | **P** |
| --- | --- | --- |
| Phase of COVID-19 pandemic: - before first lockdown (1 January 2019 to 21 March 2020) - during first lockdown (22 March 2020 to 6 May 2020) - after first lockdown (7 May 2020 to 12 December 2020) - during second lockdown (13 December 2020 to 30 June 2021) - after second lockdown (1 July 2021 to 31 December 2021) | reference 0.21 (0.11/0.31) 0.23 (0.18/0.28) 0.40 (0.33/0.47) 0.27 (0.18/0.36) | <0.001 <0.001 <0.001 <0.001 |
| Measurement number | -0.03 (-0.04/-0.02) | <0.001 |
| HbA1c at previous measurement | 0.34 (0.32/0.36) | <0.001 |
| Diabetes medication in the 90 days before HbA1c measurement: - no medication - oral medication, but no GLP1 analogues and no insulins - oral medication and GLP1 analogues, but no insulins - intermediate- or long-acting insulins, but no fast-acting or mixed - fast-acting or mixed insulins | reference 0.09 (0.03/0.14) -0.02 (-0.10/0.06) 0.24 (0.16/0.32) 0.31 (0.25/0.38) | 0.003 0.614 <0.001 <0.001 |

**n: number of participants; N: number of observations; CI: confidence interval**
